# Supplementary material for: p190A/ ARHGAP35 and p190B/ ARHGAP5 proteins in endometrial cancer: a novel cancer‐relevant paralog interplay
Source: Mol Oncol. 2026 Sep 10:10.1002/1878-0261.70335. Online ahead of print. doi: 10.1002/1878-0261.70335 (PMC13560762; doi:10.1002/1878-0261.70335)
Supplement: Supplementary file 1 [file MOL2-9999-0-s001.pdf]

**SUPPLEMENTARY MATERIAL**

**Supplementary Figure 1 – Screening of the HEC-1-A clones after applying the CRISPR/Cas9 technique to produce knockout of p190A (A-B) and p190B (C-D).**

(A, C) Western blots showing the expression or extinction of p190A (A) and p190B (C) proteins in the clones. Protein extract of each clone was analyzed by two independent antibodies against the targeted protein. Three clones of each KO, in which the targeted protein was absent, were chosen for the study. The clones are highlighted in blue for p190A-KO clones and in green for p190B-KO clones. They were renamed KO A-1 (clone#9), KO A-2 (clone #11) and KO A-3 (clone #16) for p190A-KO and KO B-1 (clone#7), KO B-2 (clone #9) and KO B-3 (clone #8) for p190B-KO. (B, D) The predicted sequence of each allele after Sanger chromatogram analysis is shown. The Start codon is highlighted in orange, the altered sequenced is shown in red and the PAM sequence is underlined.

**Supplementary Figure 2 – Alteration of the actin cytoskeleton upon p190A or p190B silencing.**

(A-B) F-actin alteration is due to the GAP activity of p190. HEC-1-A p190A-KO cells were transduced using lentiviral particles expressing WT or mutated (S866F, Δ865-870, R997*) p190A. (A) Expression of p190A was assessed by western-blot using anti-p190A antibodies. GAPDH was used as a loading control. Please note that p190A-R997* is a truncated version of p190A lacking the GAP domain (band at 130kDa). (B) Cells were fixed, stained with fluorescent phalloidin. The graph shows the quantification of F-Actin intensity in phalloidin-labelled cells. Mean fluorescence intensity was measured across the cells, in duplicate, with n=5 cells each time representing 10 measured points on the figure. Data are presented as mean ± SD. Result was analyzed by one-way ANOVA, **$p<0.01$, ****$p<0.0001$ when compared to the control condition. (C) Alpha-actinin colocalizes with CLANs in HEC-1-A p190 Knockout cells. Representative confocal images of HEC-1-A p190A-KO (KO A-1) and HEC-1-A p190B-KO (KO B-1) cells containing CLANs out of three independent experiments. Phalloidin (red) and α-actinin (green) staining colocalizes in the structures. Nuclei were stained using DAPI (blue). The arrows indicate CLAN nodes. Scale bar: 10 µm. (D) HEC-1-B cells form CLANs upon p190A or p190B silencing. HEC-1-B cells were transfected with siRNA targeting p190A (sip190A-3) or p190B (sip190B-1), seeded on fibronectin-coated glass coverslip, fixed and stained with fluorescent-phalloidin (F-actin, red), anti-a-actinin antibodies (green) and DAPI (nucleus, blue). Representative images of control, p190A or p190B knocked-down HEC-1-B cells. Arrows indicate CLAN structures. Scale bars: 10 µm. The graph represents the quantification of the number of cells containing CLANs. Quantification was made in triplicate with n>300 cells for each condition; data are presented as mean ± SD of three independent experiments. Result was analyzed by one-way ANOVA, *$p<0.05$, **$p<0.01$ when compared to the control condition.

__Supplementary Figure 3__ **– CLANs formation is dependent on RhoA/ROCK pathway in p190-KO HEC-1-A cells.** WT, p190A-KO and p190B-KO cells were treated or not with 10 µM of Y-27632 for 24 h. Cells were fixed, stained for F-actin and nuclei using respectively fluorescent phalloidin and DAPI. CLANs were quantified under the microscope. The graph shows the quantification of the presence of CLANs in WT and KO HEC-1-A cells. Graph is representative of three independent experiments. Data are presented as box and whiskers showing all points with Min to Max and analyzed by one-way ANOVA, *p<0.05, **p<0.01, ***p<0.001, ****p<0.0001. Result was analyzed by one-way ANOVA, *p<0.05, **p<0.01.

__Supplementary Figure 4__ **– p190A/B Knockout did not confer cisplatin resistance to HEC-1-A cells.** Cells were untreated or treated with Cisplatin (100 µM) for 72 hours. Cell proliferation was measured during treatment using the IncuCyte system. The graph presents the area under the curve of three replicates. Data are presented as mean ± SD; result was analyzed by one-way ANOVA. n.s, non-significant, ****p<0.0001.

__Supplementary Table 1:__ **Clinical and tumor characteristics of patients used in this study.** Patients are numbered from P1 to P16. Sample information is indicated as “NT(1)/T(3),” where “T” refers to tumoral tissue, “NT” to non-tumoral tissue, and the number in parentheses denotes the number of samples obtained from each patient. AWD, alive with disease; DOD, died of disease; DWD, died with disease; NED, no evidence of disease.

__Supplementary Table 2:__ **Mutational status of endometrial cell lines for *ARHGAP35* and *ARHGAP5* genes.** Data were extracted from the Cancer Cell Line Encyclopedia (CCLE) database (https://sites.broadinstitute.org/ccle/)

**A**

① **Anti-p190A Ab #1 (Clone 30, BD Transd. Lab)**

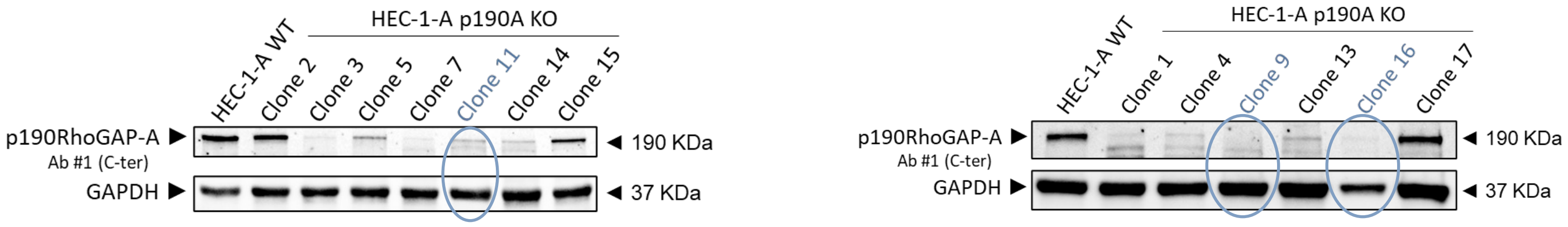


② **Anti-p190A Ab #2 (Clone D2D6, Sigma))**

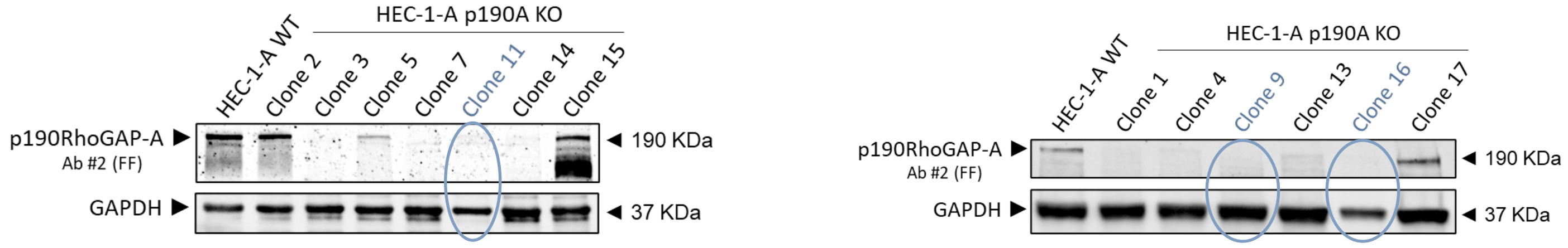


**B**

**Clone #9 (KO-A#1)**

- **Allele n°1: -1 deletion (red)**

*--ATGATGATGGCAAGAAAGCAAGATGTCCGAATTCCCACCTACAAC*
*ATCAGTGTGGTGGGATTATCTGGGACCGAGAAGGAAAAGGGCCAGTGTGGGAT*
*TGGAAAGTCTTGTTTGTGCAACCGCTT~~C~~GTGCGCCCGAGTGCTGACGAGTTTC*
*ACTTGGACCATACCTCCGTCCTCAGCACCAGTGACTTTGGAGGGCGAGTGGTC*
*AATAATGACCACTTTCTCTACTGGGGA--*

- **Allele n°2: -13 deletion (red)**

*--ATGATGATGGCAAGAAAGCAAGATGTCCGAATTCCCACCTACAAC*
*ATCAGTGTGGTGGGATTATCTGGGACCGAGAAGGAAAAGGGCCAGTGTGGGAT*
*TGGAAAGTCTTGTTTGTGC~~AACCGCTTCGTGC~~GCCCGAGTGCTGACGAGTTTC*
*ACTTGGACCATACCTCCGTCCTCAGCACCAGTGACTTTGGAGGGCGAGTGGTC*
*AATAATGACCACTTTCTCTACTGGGGA–*

**Clone #11 (KO-A#2)**

- **Allele n°1: -11 deletion (red)**

*--ATGATGATGGCAAGAAAGCAAGATGTCCGAATTCCCACCTACAAC*
*ATCAGTGTGGTGGGATTATCTGGGACCGAGAAGGAAAAGGGCCAGTGTGGGAT*
*TGGAAAGTCTTGTTTGTG~~CAACCGCTTCG~~TGCGCCCGAGTGCTGACGAGTTTC*
*ACTTGGACCATACCTCCGTCCTCAGCACCAGTGACTTTGGAGGGCGAGTGGTC*
*AATAATGACCACTTTCTCTACTGGGGA--*

- **Allele n°2: -13 deletion (red)**

*--ATGATGATGGCAAGAAAGCAAGATGTCCGAATTCCCACCTACAAC*
*ATCAGTGTGGTGGGATTATCTGGGACCGAGAAGGAAAAGGGCCAGTGTGGGAT*
*TGGAAAGTCTTGTTTGTGCAACCG~~CTTCGTGCGCCCG~~AGTGCTGACGAGTTTC*
*ACTTGGACCATACCTCCGTCCTCAGCACCAGTGACTTTGGAGGGCGAGTGGTC*
*AATAATGACCACTTTCTCTACTGGGGA–*

**Clone #16 (KO-A#3)**

- **Allele n°1: -13 deletion (red)**

*--ATGATGATGGCAAGAAAGCAAGATGTCCGAATTCCCACCTACAAC*
*ATCAGTGTGGTGGGATTATCTGGGACCGAGAAGGAAAAGGGCCAGTGTGGGAT*
*TGGAAAGTCTTGTTTGTGCAACCG~~CTTCGTGCGCCCG~~AGTGCTGACGAGTTTC*
*ACTTGGACCATACCTCCGTCCTCAGCACCAGTGACTTTGGAGGGCGAGTGGTC*
*AATAATGACCACTTTCTCTACTGGGGA--*

- **Allele n°2: -11 deletion (red)**

*--ATGATGATGGCAAGAAAGCAAGATGTCCGAATTCCCACCTACAAC*
*ATCAGTGTGGTGGGATTATCTGGGACCGAGAAGGAAAAGGGCCAGTGTGGGAT*
*TGGAAAGTCTTGTTTGTG~~CAACCGCTTCG~~TGCGCCCGAGTGCTGACGAGTTTC*
*ACTTGGACCATACCTCCGTCCTCAGCACCAGTGACTTTGGAGGGCGAGTGGTC*
*AATAATGACCACTTTCTCTACTGGGGA–*

**Figure S1**

**C**

## ① Anti-p190B Ab #1 (Clone 54, BD Transd. Lab.))

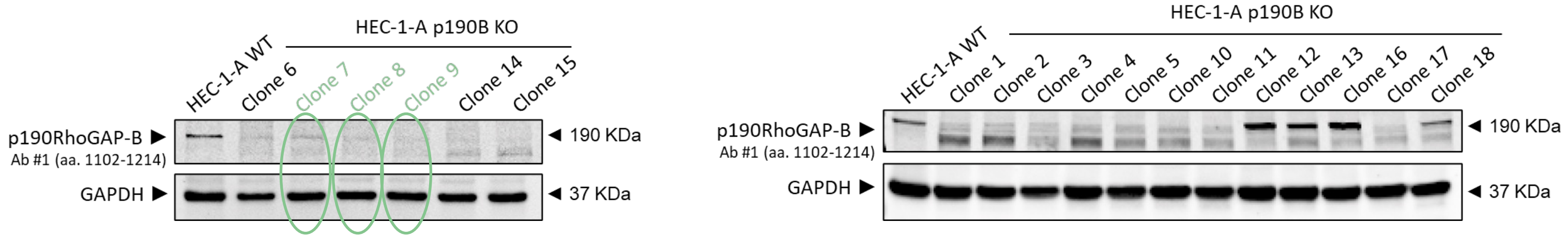


## ② Anti-p190B Ab #2 (polyclonal, Cell Signaling Tech.)

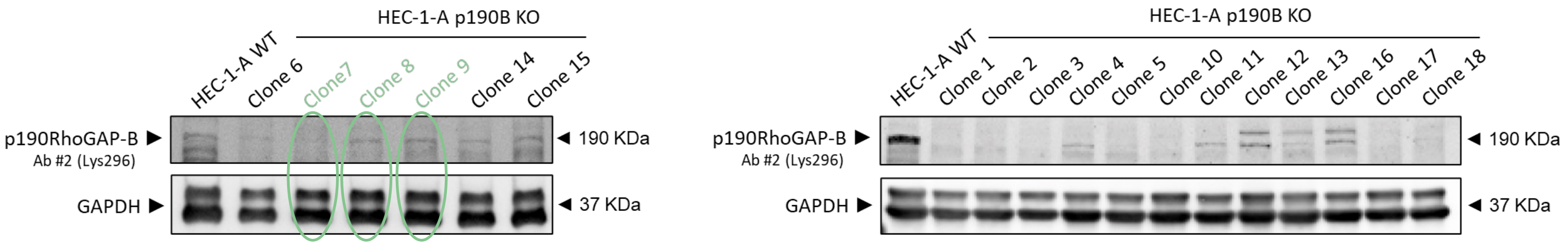


**D**

### Clone #7 (KO-B#1)

- **Allele n°1: +1 insertion (green)**

*--ATGATGGCAAAAAACAAAGAGCCTCGTCCCCCATCCCTAT*
*ACCATCAGTATAGTTGGACTCTCTGGGACTGAAAAAGACAAAGGTAACTGTGG*
*AGTTGGAAAGTCTTGTTTGTGCAATAGATTTGTACGCTCA–*

- **Allele n°2: -10 deletion (red)**

*--ATGATGGCAAAAAACAAAGAGCCTCGTCCCCCATC~~CTAT~~*
*~~ACCATC~~AGTATAGTTGGACTCTCTGGGACTGAAAAAGACAAAGGTAACTGTGG*
*AGTTGGAAAGTCTTGTTTGTGCAATAGATTTGTACGCTCA–*

### Clone #9 (KO-B#2)

- **Allele n°1: +1 insertion (green)**

*--ATGATGGCAAAAAACAAAGAGCCTCGTCCCCCATCCGTAT*
*ACCATCAGTATAGTTGGACTCTCTGGGACTGAAAAAGACAAAGGTAACTGTGG*
*AGTTGGAAAGTCTTGTTTGTGCAATAGATTTGTACGCTCA–*

- **Allele n°2: -10 deletion (red)**

*--ATGATGGCAAAAAACAAAGAGCCTCGTCCCCCATC~~CTAT~~*
*~~ACCATC~~AGTATAGTTGGACTCTCTGGGACTGAAAAAGACAAAGGTAACTGTGG*
*AGTTGGAAAGTCTTGTTTGTGCAATAGATTTGTACGCTCA–*

### Clone #8 (KO-B#3)

- **Allele n°1: -10 deletion (red)**

*--ATGATGGCAAAAAACAAAGAGCCTCGTCCCCCATC~~CTAT~~*
*~~ACCATC~~AGTATAGTTGGACTCTCTGGGACTGAAAAAGACAAAGGTAACTGTGG*
*AGTTGGAAAGTCTTGTTTGTGCAATAGATTTGTACGCTCA–*

- **Allele n°2: -2 deletion (red)**

*--ATGATGGCAAAAAACAAAGAGCCTCGTCCCCCATC~~CT~~AT*
*ACCATCAGTATAGTTGGACTCTCTGGGACTGAAAAAGACAAAGGTAACTGTGG*
*AGTTGGAAAGTCTTGTTTGTGCAATAGATTTGTACGCTCA–*

**Figure S1**

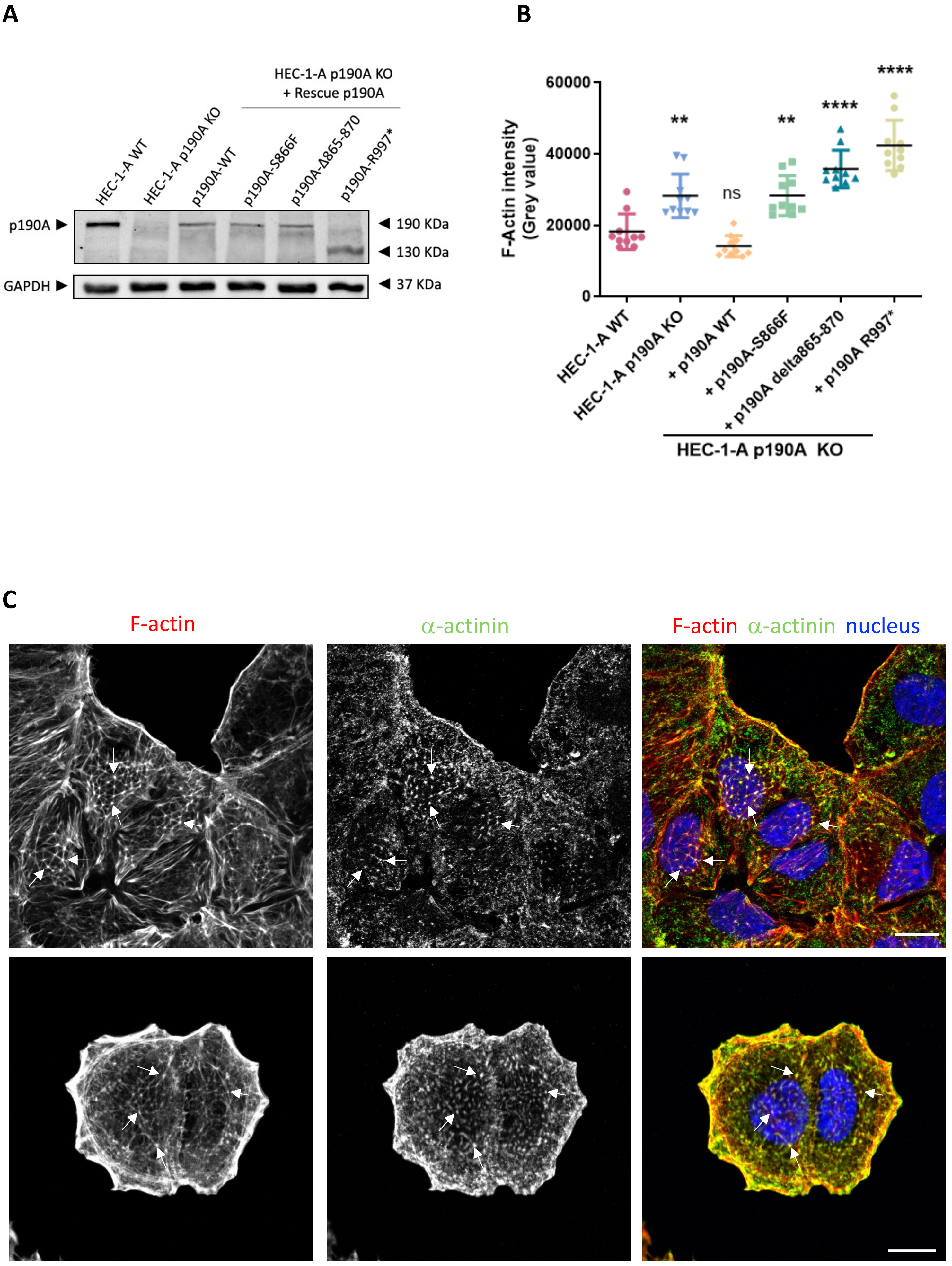
A
HEC-1-A p190A KO
+ Rescue p190A
HEC-1-A WT
HEC-1-A p190A KO
p190A-WT
p190A-S866F
p190A-Δ865-870
p190A-R997*
p190A
GAPDH
190 KDa
130 KDa
37 KDa
B
F-Actin intensity
(Grey value)
60000
40000
20000
0
**
ns
**
****
****
HEC-1-A WT
HEC-1-A p190A KO
+ p190A WT
+ p190A-S866F
+ p190A delta865-870
+ p190A R997*
HEC-1-A p190A KO
C
F-actin
α-actinin
F-actin α-actinin nucleus
HEC-1-A p190A KO
HEC-1-A p190B KO

# Figure S2

D

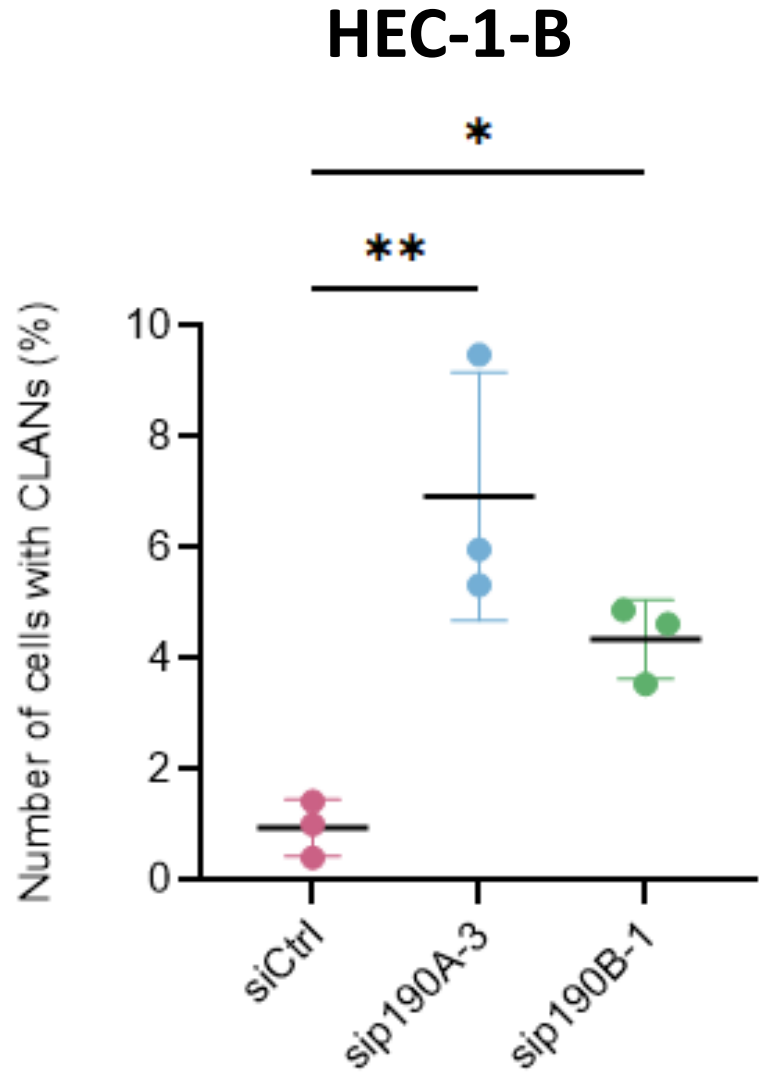
HEC-1-B
*
**
Number of cells with CLANs (%)
10
8
6
4
2
0
siCtrl
sip190A-3
sip190B-1

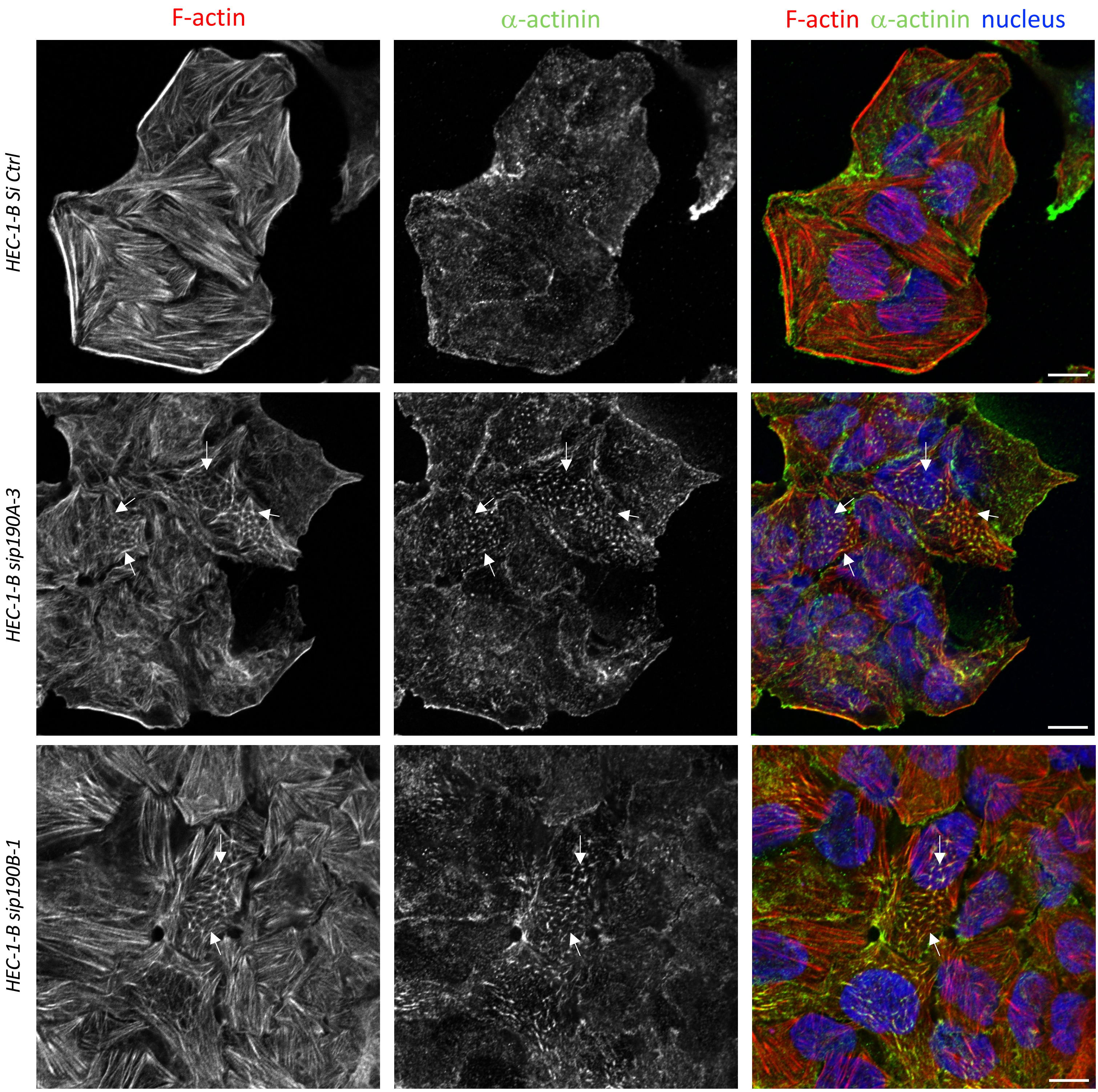
F-actin
α-actinin
F-actin α-actinin nucleus
HEC-1-B Si Ctrl
HEC-1-B sip190A-3
HEC-1-B sip190B-1

Figure S2

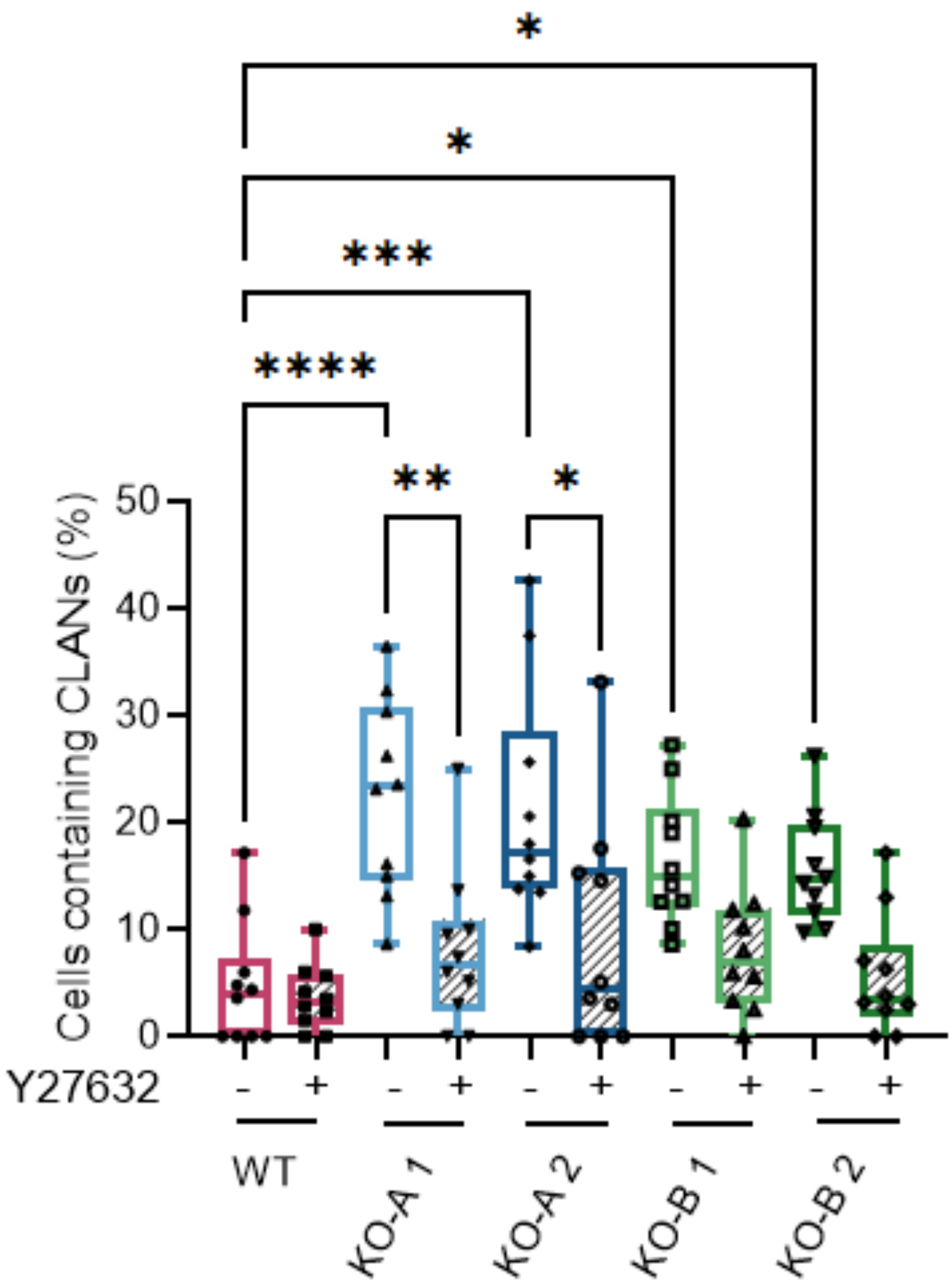

*
*
***
****
**
*
Cells containing CLANs (%)
50
40
30
20
10
0
Y27632
-
+
-
+
-
+
-
+
-
+
WT
KO-A 1
KO-A 2
KO-B 1
KO-B 2


**Figure S3**

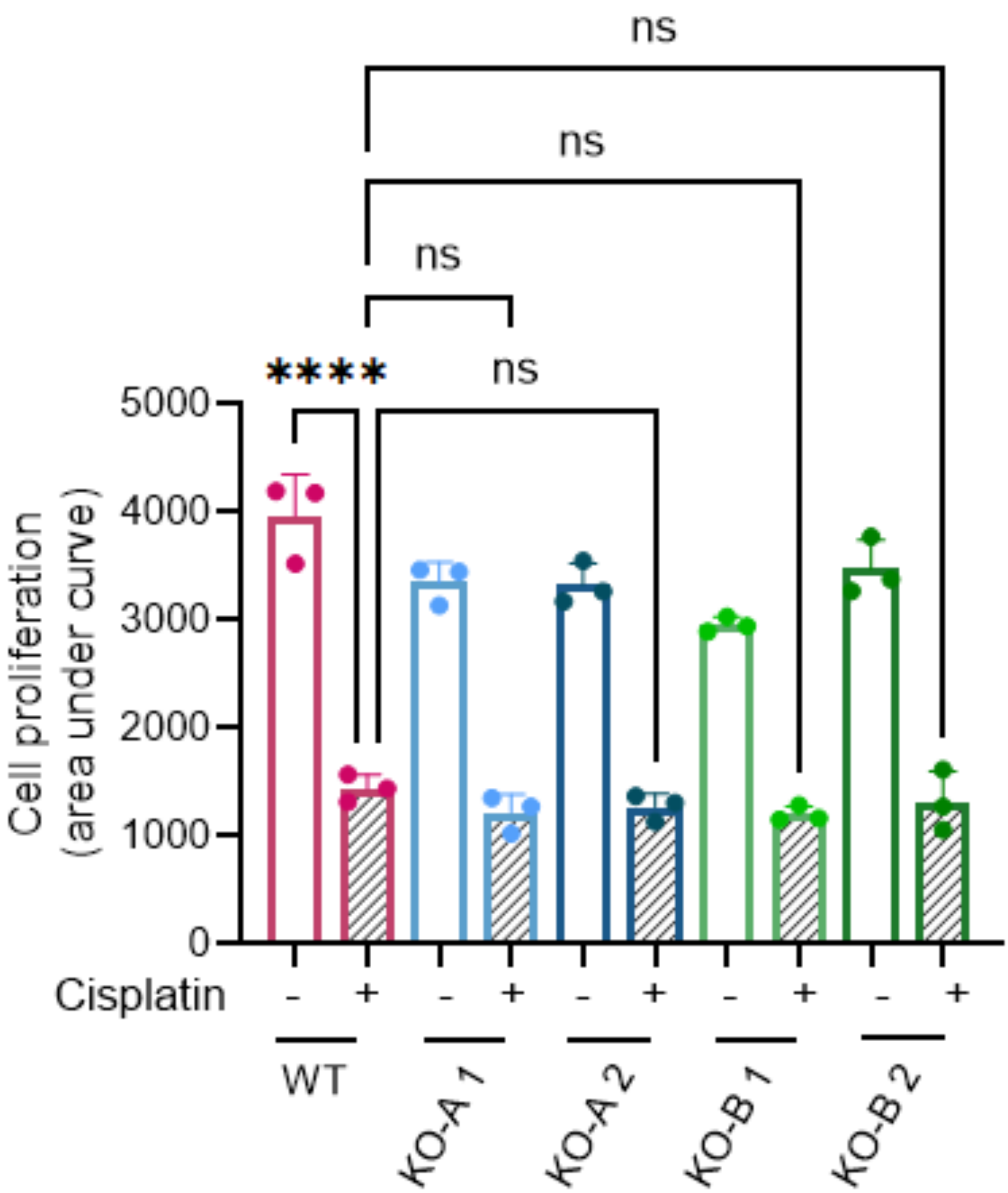
ns
ns
ns
****
ns
5000
4000
3000
2000
1000
0
Cell proliferation
(area under curve)
Cisplatin
- + - + - + - + - +
WT
KO-A 1
KO-A 2
KO-B 1
KO-B 2

**Figure S4**

**<u>Supplementary Table 1:</u> Clinical and tumor characteristics of the patients included in this study.** Patients are numbered from P1 to P16. Sample information is indicated as "NT(1)/T(3)," where "T" refers to tumoral tissue, "NT" to non-tumoral tissue, and the number in parentheses denotes the number of samples obtained from each patient. AWD, alive with disease; DOD, died of disease; DWD, died with disease; NED, no evidence of disease.

| Patient | Samples | Cancer type | Stage | Grade | % of normal cells in NT sample | % of cancer cells in T sample | Follow Up | Age |
|---|---|---|---|---|---|---|---|---|
| **P1** | T(1) | Undifferenciated | FIGO stage IA | grade 3 | | 40 | NED | 72 |
| **P2** | NT(1)/T(3) | Endometrioid | FIGO stage II | grade 2 | 60 | 60/70/60 | NED | 44 |
| **P3** | NT(1)/T(1) | Clear cell | FIGO stage IIIC | grade 3 | 20 | 40 | DWD | 78 |
| **P4** | T(3) | Endometrioid | FIGO stage II | grade 2 | | 60/60/50 | NED | 65 |
| **P5** | T(1) | Endometrioid | FIGO stage IA | grade 2 | | 60 | NED | 70 |
| **P6** | NT(1)/T(1) | Serous | FIGO stage IVB | grade 3 | 30 | 70 | DWD | |
| **P7** | T(1) | Endometrioid | FIGO stage IIIC | grade 3 | | 40 | T | |
| **P8** | NT(1)/T(1) | Endometrioid | FIGO stage IB | grade 1 | 30 | 30 | NED | |
| **P9** | NT(1)/T(1) | Endometrioid | FIGO stage IA | grade 1 | 20 | 0 | DOD | |
| **P10** | NT(1)/T(1) | Serous | FIGO stage IB | grade 2 | 60 | 60 | T | |
| **P11** | NT(1)/T(3) | Serous | FIGO stage IA | | 60 | 40/40/60 | NED | |
| **P12** | NT(1)/T(2) | Endometrioid | FIGO stage IB | grade 3 | 50 | 60/60 | T | |
| **P13** | NT(1)/T(1) | Serous | FIGO stage IA | | 40 | 30 | DOD | |
| **P14** | NT(1)/T(1) | Endometrioid | FIGO stage IA | grade 1 | 60 | 50 | NED | |
| **P15** | NT(1)/T(1) | Serous | FIGO stage IV | grade 3 | 60 | 70 | AWD | |
| **P16** | NT(1)/T(3) | Serous | FIGO stage IVB | | 40 | 70/70/70/70 | AWD | |

**<u>Supplementary Table 2</u>: Mutational status of endometrial cell lines for *ARHGAP35* and *ARHGAP5* genes.** Data were extracted from the Cancer Cell Line Encyclopedia (CCLE) database (https://sites.broadinstitute.org/ccle/)

| **Cell lines** | ***ARHGAP35*** | ***ARHGAP5*** |
|---|---|---|
| AN3CA | WT | p.P969H (Missense mutation) |
| HEC-1-A | p.K35E (Missense)<br>p.L351R (missense) | WT |
| HEC-1-B | p.L351R (Missense mutation) | WT |
| Ishikawa | WT | p.F935fs (Frameshift) |
| KLE | WT | p.A1357A (Silent) |
| RL95-2 | WT | p.N155N (Silent)<br>p.F965F (Silent)<br>p.R955S (Missense) |
